# A High-Throughput Strategy for Dissecting Mammalian Genetic Interactions

**DOI:** 10.1371/journal.pone.0167617

**Published:** 2016-12-09

**Authors:** Victoria B. Stockman, Lila Ghamsari, Gorka Lasso, Barry Honig, Sagi D. Shapira, Harris H. Wang

**Affiliations:** 1 Department of Systems Biology, Columbia University Medical Center, New York, New York, United States of America; 2 Department of Microbiology and Immunology, Columbia University Medical Center, New York, New York, United States of America; 3 Department of Biochemistry, Columbia University Medical Center, New York, New York, United States of America; 4 Department of Pathology and Cell Biology, Columbia University Medical Center, New York, New York, United States of America; Mayo Clinic, UNITED STATES

## Abstract

Comprehensive delineation of complex cellular networks requires high-throughput interrogation of genetic interactions. To address this challenge, we describe the development of a multiplex combinatorial strategy to assess pairwise genetic interactions using CRISPR-Cas9 genome editing and next-generation sequencing. We characterize the performance of combinatorial genome editing and analysis using different promoter and gRNA designs and identified regions of the chimeric RNA that are compatible with next-generation sequencing preparation and quantification. This approach is an important step towards elucidating genetic networks relevant to human diseases and the development of more efficient Cas9-based therapeutics.

## Introduction

Complex cellular processes that control cell state and decision-making are orchestrated through highly interconnected regulatory networks. Quantitative genetic interaction mapping enables the systematic discovery of how gene-gene interactions give rise to complex cellular processes [1]. Uncovering genetic interactions in lower organisms has led to novel insights into network topology and discovery of unexpected relationships between network components [2–4]. However, delineating these interactions has been largely elusive in mammalian systems due to a lack of robust experimental tools.

The CRISPR-Cas9 system enables efficient genome engineering of mammalian cells through a programmable guide-RNA (gRNA) that targets Cas9 to a desired locus for editing [5–8]. Thus far, studies using this system have focused on editing single loci [9–12] or multiple targets in select cases [13–15]. Recently, the CombiGEM approach was described to generate combinatorial gRNA libraries [16]. However, the approach requires iterative cloning steps and additional barcoding sequences. To extend CRISPR-Cas9 approaches for high-throughput combinatorial studies of genetic interactions, a general strategy is needed to interrogate pairs of chromosomal loci in a streamlined systematic and facile manner. Here, we describe the development of a multiplex strategy for assessing genetic interactions using CRISPR-Cas9 (MoSAIC).

## Materials and Methods

### Cell Culture

HEK 293T cells were obtained from the American Tissue Collection Center (ATCC) and grown at 37°C, 5% CO_2_ in high-Glucose Dulbecco’s modified Eagle’s medium (DMEM) containing 10% fetal bovine serum and 1% Penicillin/Streptomycin (Life Technologies). HEK 293T cells containing eGFP were a gift from Stephen Goff (Columbia University). 293FT cells were obtained from Life Technologies and were maintained in the same medium formulation and supplemented with 0.1 mM non-essential amino acids, 2 mM L-glutamine and 500 ug/ml Geneticin.

### Lentivirus Production and Transduction

Lentivirus was produced in 293FT cells and stable Cas9-eGFP cells were transduced as previously described (Broad Institute RNAi Consortium; http://www.broadinstitute.org/rnai/public/resources/protocols).

### Generation of inducible eGFP-Cas9 Cell Line

Briefly, doxycycline hyclate (Sigma) inducible Cas9 cells were generated as follows. 293T cell clones stably expressing eGFP-Cas9 under dox inducible promoter were generated by transduction of PLX301-eGFP-Cas9/Bsd (based on pCW-Cas9 construct, Addgene 50661) using LT1 transfection reagent (Mirus) followed by selection with 10mg/ml Blasticidin (Bsd). 293T cells were infected with lentiviral particles at MOI of 0.3 followed by clonal selection. We selected a clone with highest differential Cas9 expression following 48 hour induction using immunostaining of FLAG-tagged Cas9, followed by flow cytometry.

### Knockout Efficiency Measurements

The eGFP-Cas-9 clone was infected with lentivirus containing gRNA constructs targeting eGFP and STAT1 or eGFP-only. Twenty-four hours post-infection, the media was changed and supplemented with 10 ug/ml blasticidin (Life Technologies) and cells were selected for three days, prior to doxycycline induction of Cas9. Cells were harvested on days 14, 21, and 28 post-induction. Gene knockout efficiencies were measured by either flow cytometry or SURVEYOR assay. Flow cytometry was performed using a LSRII or LSR Fortessa to quantify fraction of eGFP positive cells.

### MoSAIC Vector Construction

MV.1, MV.3, MV.5, MV.6, MV.7 originated from lentivector v_w0, originally called plxsgRNA (Addgene 50662). A point mutation was made in the PGK promoter to eliminate the BsmB1 restriction site for all down-stream cloning (v_w0).

MV.2 originated from pLenticrispr (49535). Vs.d1 was amplified with primers containing eGFP gRNA 1 / STAT1 gRNA2 and cloned into the pLenticrispr vector to generate an all-in-one vector containing two gRNAs.

To clone MV.1 backbone, pLenticrispr was used as a template with vs_p39(f) and vs_p40 (r) to amplify an insert containing the reverse direction chimeric RNA, filler region with BsmB1 restriction sites and a forward direction chimeric RNA sequence. The chimeric- filler-chimeric was cloned into v_w0. To clone in gRNAs, vs.d5 (dsDNA) containing reverse direction H1 promoter, LoxP site and forward direction U6 promoter, was amplified with primers containing eGFP gRNA 1 and STAT1 gRNA 2 as well as BsmB1 restriction sites. The PCR product containing both gRNAs and both promoters was cloned into the MV.1 backbone to generate MV.1.1 and MV.1.2.

To clone MV.3 backbone, H1 promoter expressing short tracr RNA was cloned into v_w0 from px261 (Addgene 42337). To clone in gRNAs, vs.d11 (containing U6 promoter) was amplified with primers vs_p79 and vs_p80/ vs_p81 / vs_p82 and PCR products were cloned into MV.3 backbone.

To clone MV.5 backbone, the U6 promoter, filler region and chimeric RNA was cloned into v_w0 from lenticrispr_v1 (Addgene #49535) using vs_p59/vs_p40 primers. To clone in gRNAs,v_w2 containing chimeric RNA-LoxP-site and H1 promoter were amplified with primers containing eGFP gRNA 1 and STAT1 gRNA 2 as well as BsmB1 restriction sites.

To clone MV.6 backbone, U6 promoter, the filler region with BsmB1 restriction sites and the chimeric RNA v2, was cloned into v_w0 using vs_d10. To clone in gRNAs,v_w2 containing chimeric RNA-loxP-site and H1 promoter were amplified with primers containing eGFP gRNA 1 and STAT1 gRNA 2 as well as BsmB1 restriction sites.

In MV.7, used the backbone established in MV.6. To clone in gRNAs, oligo pairs (vs_p75 / vs_p76 and vs_p77/vs_p78) containing -BsmB1 Overhang-gRNA1-chimeric RNA-gRNA 2 were synthesized, annealed and ligated into backbone.

### DNA Constructs Used

Unless noted, all DNA constructs and primers were obtained from IDT (Geneblocks) and used for PCR and assembly steps as described above.

vs_d1: chimeric RNA—LoxP site—U6 promoter: GTTTTAGAGCTAGAAATAGCAAGTTAAAATAAGGCTAGTCCGTTATCAACTTGAAAAAGTGGCACCGAGTCGGTGCTTTTTTATAACTTCGTATAGCATACATTATACGAAGTTATGAGGGCCTATTTCCCATGATTCCTTCATATTTGCATATACGATACAAGGCTGTTAGAGAGATAATTAGAATTAATTTGACTGTAAACACAAAGATATTAGTACAAAATACGTGACGTAGAAAGTAATAATTTCTTGGGTAGTTTGCAGTTTTAAAATTATGTTTTAAAATGGACTATCATATGCTTACCGTAACTTGAAAGTATTTCGATTTCTTGGCTTTATATATCTTGTGGAAAGGACGAAACACC

vs_d5: reverse H1 promoter—LoxP site—U6 promoter forward: TAGATCTGTGGTCTCATACAGAACTTATAAGATTCCCAAATCCAAAGACATTTCACGTTTATGGTGATTTCCCAGAACACATAGCGACATGCAAATATTGCAGGGCGCCACTCCCCTGTCCCTCACAGCCATCTTCCTGCCAGGGCGCACGCGCGCTGGGTGTTCCCGCCTAGTGACACTGGGCCCGCGATTCCTTGGAGCGGGTTGATGACGTCAGCGTTCGAATTATAACTTCGTATAGCATACATTATACGAAGTTATGAGGGCCTATTTCCCATGATTCCTTCATATTTGCATATACGATACAAGGCTGTTAGAGAGATAATTAGAATTAATTTGACTGTAAACACAAAGATATTAGTACAAAATACGTGACGTAGAAAGTAATAATTTCTTGGGTAGTTTGCAGTTTTAAAATTATGTTTTAAAATGGACTATCATATGCTTACCGTAACTTGAAAGTATTTCGATTTCTTGGCTTTATATATCTTGTGGAAAGGACGAAACACC

v_w2: chimeric RNA—LoxP site—forward H1 promoter: GTTTTAGAGCTAGAAATAGCAAGTTAAAATAAGGCTAGTCCGTTATCAACTTGAAAAAGTGGCACCGAGTCGGTGCTTTTTTATAACTTCGTATAGCATACATTATACGAAGTTATAATTCGAACGCTGACGTCATCAACCCGCTCCAAGGAATCGCGGGCCCAGTGTCACTAGGCGGGAACACCCAGCGCGCGTGCGCCCTGGCAGGAAGATGGCTGTGAGGGACAGGGGAGTGGCGCCCTGCAATATTTGCATGTCGCTATGTGTTCTGGGAAATCACCATAAACGTGAAATGTCTTTGGATTTGGGAATCTTATAAGTTCTGTATGAGACCACAGATCTA

vs_d10: Xho1 site—forward U6 promoter—BsmB1 filler region (36nt)—Chimeric RNA version 2—Nhe1: ATTCGAACTCGAGGAGGGCCTATTTCCCATGATTCCTTCATATTTGCATATACGATACAAGGCTGTTAGAGAGATAATTAGAATTAATTTGACTGTAAACACAAAGATATTAGTACAAAATACGTGACGTAGAAAGTAATAATTTCTTGGGTAGTTTGCAGTTTTAAAATTATGTTTTAAAATGGACTATCATATGCTTACCGTAACTTGAAAGTATTTCGATTTCTTGGCTTTATATATCTTGTGGAAAGGACGAAACACCGGAGACGGTTTTCTTGCTCTTTTTTGTACGTCTCTGTTTTAGAGCCGGAAACGGCAAGTTAAAATAAGGCTAGTCCGTTATCAACTTGAAAAAGTGGCACCGAGTCGGTGCTTTTTTGCTAGCGCTAAC

vs_d11: forward U6 promoter: GAGGGCCTATTTCCCATGATTCCTTCATATTTGCATATACGATACAAGGCTGTTAGAGAGATAATTAGAATTAATTTGACTGTAAACACAAAGATATTAGTACAAAATACGTGACGTAGAAAGTAATAATTTCTTGGGTAGTTTGCAGTTTTAAAATTATGTTTTAAAATGGACTATCATATGCTTACCGTAACTTGAAAGTATTTCGATTTCTTGGCTTTATATATCTTGTGGAAAGGACGAAACACC

### Primers Used

vs_p26 (forward sequencing primer, all): AATGGACTATCATATGCTTACCGTAACTTGAAAGTATTTCG

vs_p64 (reverse sequencing primer, MV.6 / MV.7): TATTTTAACTTGCCGTTTCCGGC

vs_p59 (forward): ACGGACTCGAGGAGGGCCTATTTCCCATGATTC

vs_p40 (reverse): GATCACGGAGCTAGCCTGCCATTTGTCTCAAGATCTAGAATTC

vs_p75: CACCGGAGCTGGACGGCGACGTAAAGTTTTAGAGCTAGAAATAGCAAGTTAAAATAAGGCTAGTCCGTTATCAACTTGAAAAAGTGGCACCGAGTCGGTGCTTGAAGTTCGAGGGCGACACCC

vs_p76: AAACGGGTGTCGCCCTCGAACTTCAAGCACCGACTCGGTGCCACTTTTTCAAGTTGATAACGGACTAGCCTTATTTTAACTTGCTATTTCTAGCTCTAAAACTTTACGTCGCCGTCCAGCTCC

vs_p77: CACCGGAGCTGGACGGCGACGTAAAGTTTTAGAGCTAGAAATAGCAAGTTAAAATAAGGCTAGTCCGTTATCAACTTGAAAAAGTGGCACCGAGTCGGTGCTTTCCCCGGGGAAGTTCGAGGGCGACACCC

vs_p78: AAACGGGTGTCGCCCTCGAACTTCCCCGGGGAAAGCACCGACTCGGTGCCACTTTTTCAAGTTGATAACGGACTAGCCTTATTTTAACTTGCTATTTCTAGCTCTAAAACTTTACGTCGCCGTCCAGCTCC

vs_p79 (forward): AGGGATCCTGAGGGCCTATTTCCCATGA

vs_p80 (reverse): GCGCTAGCTAAAAACAGCATAGCTCTAAAACGGGTGTCGCCCTCGAACTTCACAGCATAGCTCTAAAACTTTACGTCGCCGTCCAGCTCGGTGTTTCGTCCTTTCCACAA

vs_p81 (reverse): TTAGCGCTAGCTAAAAGTTTTGGGACCATTCAAAACAGCATAGCTCTAAAACGGGTGTCGCCCTCGAACTTCGTTTTGGGACCATTCAAAACAGCATAGCTCTAAAACTTTACGTCGCCGTCCAGCTCGGTGTTTCGTCCTTTCCACAAG

vs_p82 (reverse): AGCGCTAGCTAAAAGTTTTGGGACCATTCAAAACAGCATAGCTCTAAAACGGGTGTCGCCCTCGAACTTCGTTTTGGGACCATTCAAAACAGCATAGCTCTAAAACTTTACGTCGCCGTCCAGCTCGTTTTGGGACCATTCAAAACAGCATAGCTCTAAAACGGTGTTTCGTCCTTTCCACA

### gRNA Sequence Used

*eGFP gRNA 1*: GAGCTGGACGGCGACGTAAA

*eGFP gRNA 2*: GAAGTTCGAGGGCGACACCC

*STAT1 gRNA 1*: GATCATCCAGCTGTGACAGG

*STAT1 gRNA 2*: CCTGTCACAGCTGGATGATC

*eGFP sequence*: ATGGTGAGCAAGGGCGAGGAGCTGTTCACCGGGGTGGTGCCCATCCTGGTCGAGCTGGACGGCGACGTAAACGGCCACAAGTTCAGCGTGTCCGGCGAGGGCGAGGGCGATGCCACCTACGGCAAGCTGACCCTGAAGTTCATCTGCACCACCGGCAAGCTGCCCGTGCCCTGGCCCACCCTCGTGACCACCCTGACCTACGGCGTGCAGTGCTTCAGCCGCTACCCCGACCACATGAAGCAGCACGACTTCTTCAAGTCCGCCATGCCCGAAGGCTACGTCCAGGAGCGCACCATCTTCTTCAAGGACGACGGCAACTACAAGACCCGCGCCGAGGTGAAGTTCGAGGGCGACACCCTGGTGAACCGCATCGAGCTGAAGGGCATCGACTTCAAGGAGGACGGCAACATCCTGGGGCACAAGCTGGAGTACAACTACAACAGCCACAACGTCTATATCATGGCCGACAAGCAGAAGAACGGCATCAAGGTGAACTTCAAGATCCGCCACAACATCGAGGACGGCAGCGTGCAGCTCGCCGACCACTACCAGCAGAACACCCCCATCGGCGACGGCCCCGTGCTGCTGCCCGACAACCACTACCTGAGCACCCAGTCCGCCCTGAGCAAAGACCCCAACGAGAAGCGCGATCACATGGTCCTGCTGGAGTTCGTGACCGCCGCCGGGATCACTCTCGGCATGGACGAGCTGTACAAGTAA

### Modeling of Cas9-gRNA-chimeric-RNAv2 Complex

We used the crystal structure of the sgRNA-targetDNA-cas9 complex to model the UA31CG-AU32GC-sgRNA [17]. The corresponding base pairs (U31-A38 and A32-U37) were mutated with 3DNA keeping the sugar-phosphate backbone conformation and the base reference frame as in the crystal structure [18]. The mutated RNA structure was then locally minimized with NAMD through the autoIMD plugin [19]. Atoms from mutated nucleotides were free to move while atoms within 8 Å of any mutated nucleotide atom were fixed and the remaining atoms were excluded during minimization. Conjugate gradient minimization was carried out using the CHARMM27 forcefield during 15,000 steps.

## Results

**Fig 1:**
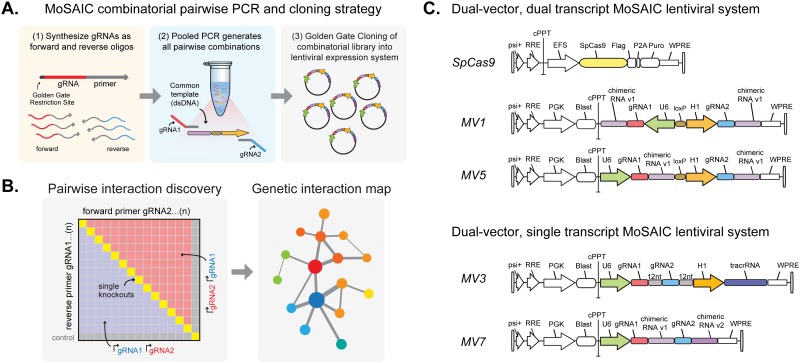
MoSAIC, a multiplex strategy for assessing genetic interactions using CRISPR-Cas9. (A) General strategy for generation of a combinatorial gRNA library targeting loci in a pairwise fashion. (B) Systematic pairwise interaction characterizations enabled by MoSAIC libraries contain gRNAs in both positions and single knockout controls. (C) Design of MoSAIC lentiviral expression systems for dual-transcript and single-transcript gRNAs.

In order to comprehensively map genetic interactions in a gene network, all possible single and double knockouts (KO) need to be simultaneously interrogated. MoSAIC achieves this in a single step through PCR of a common DNA template with gRNA primer pools (Fig 1A). The first position gRNAs act as the forward primers while the second position gRNAs act as the reverse primers. The pooled PCR product is then cloned into a lentiviral expression vector resulting in an exhaustive combinatorial dual-gRNA library. In addition to directing genome editing to the desired targets, lentiviral integration of each gRNA pair in the library serves as a unique molecular barcode of each mutant for subsequent multiplex interrogation of the cell population (Fig 1B).

To optimize the system for simultaneous targeting of Cas9 to multiple loci, we designed and tested two MoSAIC-compatible strategies:1) dual promoter, dual gRNA transcripts, and 2) single promoter, single RNA transcript (dual gRNA fusion). We explored several designs that use RNA Pol III promoters U6 and H1 in different positions and orientations (Fig 1C), having eliminated designs where the common templates contain sequences that result in DNA hairpins; for example, inward facing promoters would necessitate a common template containing two complimentary chimeric RNAs. After lentiviral transduction of MoSAIC designs into HEK293T tet-inducible Cas9 cells containing an integrated eGFP, we monitored Cas9-mediated eGFP KO by flow-cytometry 14 and 21 days post Cas9 induction (see Materials and Methods).

**Fig 2:**
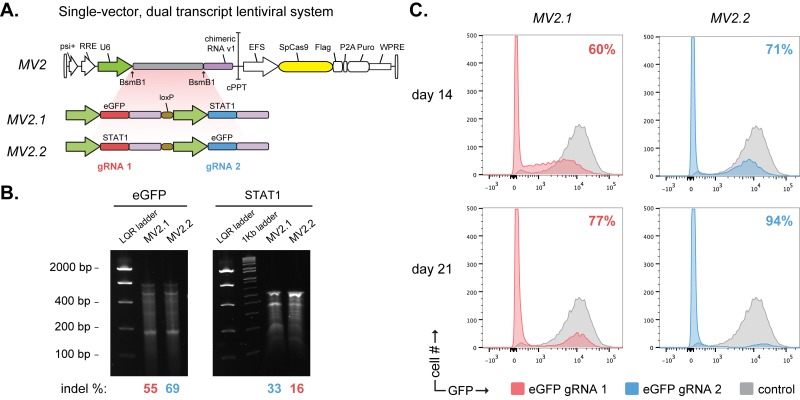
Characterization of dual U6 promoter MoSAIC designs. (A) Dual U6 promoter design tested in HEK293T, eGFP+ cells using an all-in-one vector, pLentcrispr_v1 containing both Cas9 and dual gRNA expression cassette. (B) SURVEYOR assay demonstrating positional bias at both eGFP and STAT1 targeted loci. MV2 constructs used STAT1_gRNA_1. Indel efficiencies are indicated for gRNA_1 and gRNA_2 positions below the gel in red and blue respectively. (C) Flow cytometry data at day 14 and 21 post viral transduction and puromycin selection. Indicated on the top-right of each plot are percentage KO.

We began by benchmarking a previously described approach utilizing two unidirectional U6 promoters to express dual gRNAs [13] (designs MV2; Fig 2A). We found that gRNAs expressed from the first U6 position resulted in lower efficiency than those expressed from the second position, irrespective of the target gene. Targeted KO efficiency for eGFP was determined by SURVEYOR assay to be 55% and 69% for the first and second gRNA positions respectively after 14 days (Fig 2B). Similarly, KO efficiency for STAT1 was 16% and 33% for the first and second gRNA positions. Flow cytometry measurements of eGFP KO at day 14 show consistent trends of higher second gRNA position KO efficiency. The positional KO efficiency bias persists even as the overall KO efficiency improves for both positions beyond day 21 (Fig 2C).

**Fig 3:**
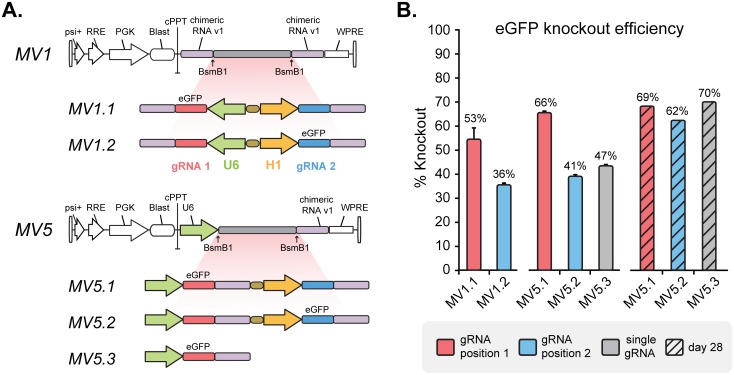
Characterization of U6-H1 dual promoter MoSAIC designs. (A) To test positional efficiency, eGFP gRNA_1 was placed in either position 1 (MV1.1, Mv5.1) or position 2 (MV1.2, MV5.2) and cloned into lentiviral vectors for testing. Single gRNA vectors were tested (MV5.3) in comparison. (B) Positional eGFP knockout efficiency for various U6-H1 dual promoter designs was determined by flow cytometric analysis of eGFP expression at day 14 (solid bars). A separate experiment was done and eGFP KO efficiency was determined at day 28 (shaded bars). Data shown with error-bars are averages from three independent experiments. Error-bars represent standard-errors of mean (n = 3, for day 28 n = 1).

We then explored whether the promoter choice and orientation impacted KO efficiency in a position-dependent fashion (designs MV1 and MV5; Fig 3A). For the bidirectional U6-H1 design (MV1), the first gRNA position driven by the U6 promoter showed higher efficiency compared to the second gRNA position driven by the H1 promoter (53% vs. 36%; Fig 3B). For the unidirectional U6-H1 design (MV5), we observed KO efficiencies of 66% and 41% at the first gRNA position (U6 promoter) and second position (H1 promoter) respectively (Fig 3B) at day 14. These results suggest that H1 promoter may be a weaker promoter (in general or transiently at day 14) and in the second gRNA position in contrast to the dual U6 promoter findings (Fig 2). However, the KO efficiencies for MV5 designs in gRNA position 1 and 2 eventually converge to 69% and 62% respectively by day 28 (Fig 3B), showing that the KO efficiency from the H1 promoter eventually reach that of the U6 promoter. The single gRNA control using the U6 promoter also reaches similar KO efficiency (70%) after 28 days. Together, these data highlight the significant impact of promoter position and orientation on KO efficiency for dual gRNA expression and suggest that the unidirectional U6-H1 design (MV5) is the most optimal implementation for targeting Cas9 to multiple loci.

**Fig 4:**
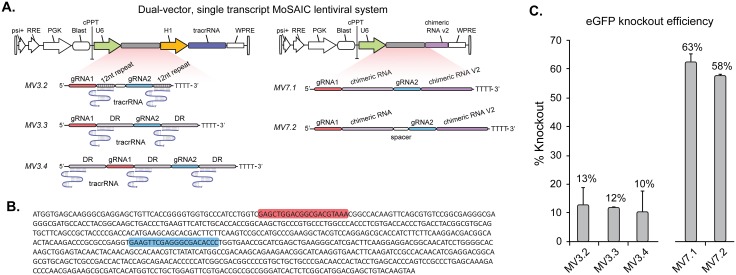
Characterization of single promoter, single transcript MoSAIC designs. (A) Schematic representation of gRNA lentivector backbone and gRNA transcript designs shown in complex with tracrRNA. MV3.2 contains only the first 12 bp of the 35 bp direct repeat sequence suggesting RNA cleavage is not necessary for Cas9 function. MV7.2 includes a spacer sequence TCCCCGGG (rationally designed to prevent hairpin formation) between gRNA sequences. gRNA1 is the eGFP gRNA_1 and gRNA 2 is eGFP gRNA_2. (B) Sequence of eGFP that was targeted using gRNA1 (red) and gRNA2 (blue). (C) Positional eGFP knockout efficiency was determined by flow cytometric analysis. All data shown are averages from three independent experiments. Error-bars represent standard-errors of mean (n = 3).

Previously, studies demonstrated that targeting Cas9 to multiple loci could be achieved by co-expressing the RNA cleavage enzyme *Csy4* along with multiplexed gRNA expression from single RNA transcripts containing RNA cleavage sites [20]. Another study observed that flanking each gRNA with *S*. *pyogenes* direct repeat (DR) sequences is sufficient for multiplexed Cas9-mediated KO in the absence of the SpRNase III RNA cleavage enzyme [15]. In order to increase the multiplexing potential of MoSAIC, we explored whether single RNA transcripts encoding multiple gRNAs can lead to efficient Cas9 targeting and gene KO. Four RNA transcript designs (each MoSAIC compatible for pairwise combinatorial library assembly) driven by a single U6 promoter and targeting two positions of an integrated eGFP gene were tested (MV3, MV7; Fig 4). In the MV3 designs, a tracrRNA was expressed separately from an H1 promoter in place of the chimeric RNA. Repeat regions between two gRNAs were altered to contain either a 12bp sequence complementary to the tracrRNA (MV3.2) or the DR sequence (MV3.3 and MV3.4) previously described [15] (Fig 4A). We observed that designs using DR sequences (MV3.3 and MV3.4) led to a limited KO efficiency (12% and 10% respectively), in accordance with previous findings. The reduced DR sequence consisting of only the 12bp repeat region (MV3.2) lead to KO efficiency on par with constructs MV3.3 and MV3.4 containing the full DR sequences (Fig 4C). While it remains to be elucidated, these results suggest that RNA cleavage of multi-gRNA transcripts is not necessary for Cas9 mediated gene editing.

We further explored MV7 designs that incorporated transcripts containing two tandem gRNA-chimeric RNA sequences and thus did not require a tracrRNA (Fig 4A). This design lead to a KO efficiency (63%) that was as good as, if not better than, the dual promoter designs (Figs 4C vs. 3B). When combined with dual U6-H1 strategies, MV7 designs may provide opportunities to increase editing efficiency (by encoding multiple gRNAs on a single transcript), as well as to reduce off-target editing (if used with nickase-Cas9) [21].

**Fig 5:**
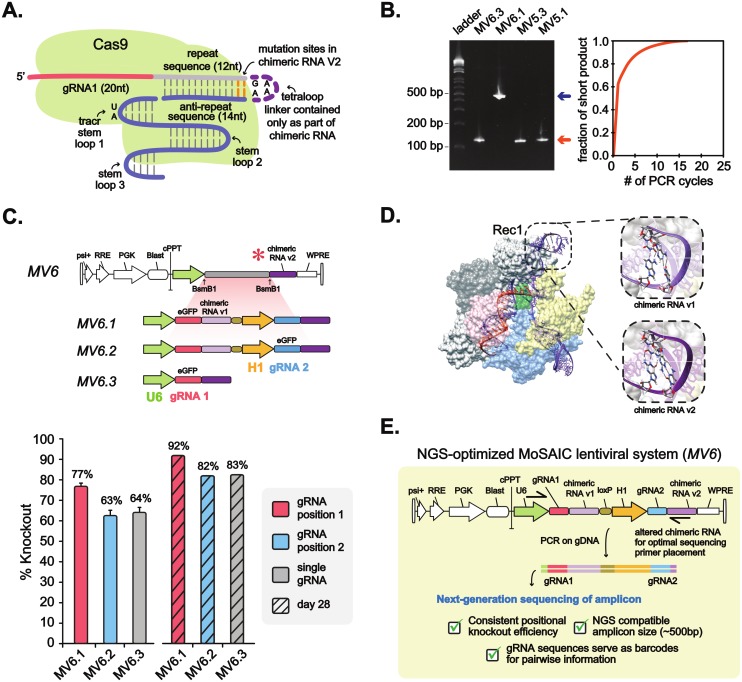
Development of a Next-Generation Sequencing optimized MoSAIC system. (A) Schematic representation of Cas9 with gRNA with sites altered highlighted in orange. (B) Recovery of gRNA library for NGS. Left panel shows PCR recovery of gRNA barcodes from genomic DNA (short product indicated by orange arrow, long product indicated by blue arrow). Right panel shows undesired enrichment of short products during PCR amplification. (C) Design and efficiency characterization of MV6, the NGS optimized MoSAIC lentiviral system, at day 14 (solid bars). A separate experiment was done to characterize efficiencies at day 28 (shaded bars). Data shown with error-bars are averages from three independent experiments. Error-bars represent standard-errors of mean (n = 3, for day 28 n = 1). (D) Crystal structure of Cas9 in complex with chimeric RNA variants. The repeat and antirepeat sequences of the chimeric RNA form an RNA duplex, which interacts with the Rec1 domain on Cas9. Alterations in chimeric RNA v2 replace T-A Watson-Crick basepairing with C-G base pairing. Base pair alterations may stabilize RNA-protein interactions, possibly increasing the efficiency of Cas9 activity. (E) Summary diagram of a lentiviral MoSAIC system for NGS quantification.

MoSAIC is designed such that gRNA pairs serve as barcodes that can then be PCR amplified and identified using next-generation sequencing. We achieved this by altering the second chimeric RNA sequence such that placement of a reverse sequencing primer results in PCR amplification of both gRNAs with an amplicon size that is NGS compatible (Fig 5A). Primer placement at repeat regions, such as two U6 promoters or two identical chimeric RNA’s leads to two potential PCR products, a long and a short, and favors the short product, which contains only one gRNA sequence (Fig 5B). We utilized the *S*. *pyogenes* CRISPR-Cas9 crystal structure (PDB-4OO8) [17] to predict mutations in the chimeric RNA sequence that would not interfere with Cas9 function and allow for optimal primer placement (see Methods). Nucleotide positions 11–12 and 17–18 (corresponding to the repeat-anti-repeat duplex flanking the tetraloop) of wild-type chimeric RNA where altered from TA----TA to CG----CG to generate an altered orthogonal chimeric RNA sequence (v2) that is compatible with PCR of gRNA barcodes. Indeed, the altered chimeric RNA enables recovery of full-length dual-gRNA barcode amplicon from extracted genomic DNA (Fig 5B). We then measured the efficiency of Cas9-mediated KO using the altered chimeric RNA designs (MV6.2/ MV6.3, Fig 5C) and found that there is a significantly higher KO efficiency than the original (MV5.2/ MV5.3) chimeric RNA (Fig 3B). This increased efficiency may be the result of tetranucleotide stabilization due to an increase in intra-strand Hydrogen-bonding, which further stabilizes Rec1-RNA interactions within Cas9 (Fig 5D), and raises the possibility that additional chimeric RNA variants may exist that lead to more efficient Cas9 editing. Importantly, these alterations enable a dual gRNA vector that is compatible with high-throughput screening (Fig 5E). The ability to utilized pooled amplification of gRNAs, which serve as molecular barcodes for individual cell variants in a population, allows for the use of next-generation sequencing to find hits in pooled cell-based screens.

## Discussion

Here we report on the development of a generalizable strategy for multiplex targeting of Cas9 for mammalian genome engineering. We describe the implementation of this approach to combinatorially target two genes simultaneously and monitor the mutational efficiency of several gRNA designs. We find that gRNA pairs expressed from dual U6-H1 promoters lead to optimal Cas9-mediated genome editing, which can be combined with single transcript multiple gRNA designs (MV7) to increase editing efficiency. In addition, we find that a unidirectional single-transcript gRNA system resulted in low KO efficiency. While the precise reason for this observation has not been fully elucidated, we speculate that MV3.3 and 3.4 designs that lead to contiguous unprocessed gRNA products may cause a lower Cas9 targeting efficiency by virtue of Cas9 binding to either of the two gRNA positions, but not simultaneously. In addition, we observed that unidirectional dual gRNA designs resulted in increased KO efficiency for the second gRNA position. While further experiments are needed, we speculate that higher KO efficiency of the second gRNA may be the result of transcription read-through of first position gRNA, leading to increased levels of second gRNA transcripts and Cas9 targeting.

To enhance multiplexing of our system, we identified specific chimeric RNA variants at positions 11–12 and 17–18 corresponding to the tetraloop repeat-anti-repeat duplex that are amenable to alteration. We showed that these chimeric RNA variants still maintained Cas9 targeting while also allowing compatible barcode amplification in a pooled format to enable multiplex assessment of cell populations using NGS. In this manner, the gRNA pairs serve as unique molecular barcodes linking their abundance in the cell population with their screened phenotype. This strategy is also compatible with the use of different Cas9 variants, including CRISPRi [22] and CRISPRa [23], enabling both loss- and gain-of-function combinatorial screens. We speculate that additional modifications to the chimeric RNA at the tetraloop region may enable further Cas9 functionality (e.g. fusion with RNA-scaffolds [24]) and believe our strategy may be of particular value for application of Cas9-nickase variants that require dual gRNAs to target a single locus [21].

MoSAIC overcomes several key technical hurdles associated with high-throughput generation and measurement of dual loci perturbations in mammalian cells. Additionally, to facilitate subsequent iterative introduction of gRNA constructs and enable higher-order combinatorial genetic perturbations, the integrated lentiviral vector design includes loxP “landing-pad” sequences. The MoSAIC system expands the toolbox for genetic modification of mammalian genome and extends our knowledge of Cas9 targeting design parameters. Recently, Wong et al described the CombiGEM approach to generate combinatorial gRNA libraries for Cas9-based genomic interrogations by barcoded sequencing [16]. The CombiGEM method utilizes iterative cloning of each gRNA with a unique pre-designed barcode into a backbone vector, followed by an additional cloning reaction to incorporate the scaffold sequence (i.e. the chimeric RNA fragment). To generate gRNA libraries with two simultaneous gRNAs, two additional cloning reactions need to be completed to add the second gRNA and its scaffold sequence. While the method conceptually enable the generation of n-wise gRNA libraries through iterative cloning reactions, in practice the library construct steps can become laborious and may introduce additional library biases due to the multiple cloning, population expansion, and library processing steps. In contrast to the four-step cloning reactions needed in ComiGEM, MoSAIC utilizes only a single cloning reaction to generate pairwise gRNA libraries. Nonetheless, additional gRNA library generation strategies will likely improve in the future. Finally, MoSAIC and similar approaches may advance the therapeutic potential of combinatorial Cas9-mediated genome editing and represents an important step towards comprehensive delineation of genetic networks relevant to human disease as well as fundamental aspects of cellular life.
